# Increased Expressions of Matrix Metalloproteinases (MMPs) in Prostate Cancer Tissues of Men with Type 2 Diabetes

**DOI:** 10.3390/biomedicines8110507

**Published:** 2020-11-16

**Authors:** Andras Franko, Lucia Berti, Jörg Hennenlotter, Steffen Rausch, Marcus O. Scharpf, Martin Hrabĕ de Angelis, Arnulf Stenzl, Andreas Peter, Andreas L. Birkenfeld, Stefan Z. Lutz, Hans-Ulrich Häring, Martin Heni

**Affiliations:** 1 Department of Internal Medicine IV, Division of Diabetology, Endocrinology and Nephrology, University Hospital Tübingen, 72076 Tübingen, Germany; andras.franko@med.uni-tuebingen.de (A.F.); andreas.birkenfeld@med.uni-tuebingen.de (A.L.B.); hans-ulrich.haering@uni-tuebingen.de (H.-U.H.); 2 Institute for Diabetes Research and Metabolic Diseases of the Helmholtz Centre Munich at the University of Tübingen, 72076 Tübingen, Germany; lucia.berti@helmholtz-muenchen.de; 3 German Center for Diabetes Research (DZD), 85764 Neuherberg, Germany; 4 Department of Urology, University Hospital Tübingen, 72076 Tübingen, Germany; joerg.hennenlotter@med.uni-tuebingen.de (J.H.); steffen.rausch@med.uni-tuebingen.de (S.R.); arnulf.stenzl@med.uni-tuebingen.de (A.S.); 5 Institute of Pathology, University Hospital Tübingen, 72076 Tübingen, Germany; marcus.scharpf@med.uni-tuebingen.de; 6 Institute of Experimental Genetics, Helmholtz Zentrum München, German Research Center for Environmental Health, 85764 Neuherberg, Germany; hrabe@helmholtz-muenchen.de; 7 Institute for Clinical Chemistry and Pathobiochemistry, Department for Diagnostic Laboratory Medicine, University Hospital Tübingen, 72076 Tübingen, Germany; andreas.peter@med.uni-tuebingen.de; 8 Clinic for Geriatric and Orthopedic Rehabilitation Bad Sebastiansweiler, 72116 Mössingen, Germany; s.lutz@bad-sebastiansweiler.de

**Keywords:** prostate cancer, diabetes, epithelial-mesenchymal transition, matrix metalloproteinase, CC chemokine ligand

## Abstract

Type 2 diabetes (T2D) is associated with worse prognosis of prostate cancer (PCa). The molecular mechanisms behind this association are still not fully understood. The aim of this study was to identify key factors, which contribute to the more aggressive PCa phenotype in patients with concurrent T2D. Therefore, we investigated benign and PCa tissue of PCa patients with and without diabetes using real time qPCR. Compared to patients without diabetes, patients with T2D showed a decreased E-cadherin/N-cadherin (CDH1/CDH2) ratio in prostate tissue, indicating a switch of epithelial-mesenchymal transition (EMT), which is a pivotal process in carcinogenesis. In addition, the gene expression levels of matrix metalloproteinases (*MMPs*) and CC chemokine ligands (*CCLs*) were higher in prostate samples of T2D patients. Next, prostate adenocarcinoma PC3 cells were treated with increasing glucose concentrations to replicate hyperglycemia in vitro. In these cells, high glucose induced expressions of *MMPs* and *CCLs*, which showed significant positive associations with the proliferation marker proliferating cell nuclear antigen (*PCNA*). These results indicate that in prostate tissue of men with T2D, hyperglycemia may induce EMT, increase *MMP* and *CCL* gene expressions, which in turn activate invasion and inflammatory processes accelerating the progression of PCa.

## 1. Introduction

The association between prostate cancer (PCa) and type 2 diabetes (T2D) is complex [1]. Many studies suggest that patients with T2D may have a reduced risk for developing PCa [2,3], however the underlying molecular mechanisms are not fully understood. One possible explanation is proposed by the reduced PCa detection in patients with diabetes due to lower prostate-specific antigen (PSA) levels compared to patients without diabetes [4]. Nevertheless, when patients with T2D are diagnosed with PCa, they are characterized by activated carcinogenic pathways [5] and suffer from more aggressive PCa [6,7] with a markedly poorer prognosis compared to patients without T2D [8,9]. Both high insulin and glucose levels are postulated to drive PCa carcinogenesis in patients with T2D [10], however the exact underlying molecular mechanisms for the accelerated PCa progression in case of coexistent T2D is not determined yet.

In this study, we aimed to identify key pathways and their regulators including epithelial-mesenchymal transition (EMT), inflammation, and invasion, which could accelerate PCa progression in patients with T2D. One of the crucial carcinogenic processes in PCa is EMT, which is defined as a switch from epithelial characteristics to mesenchymal features [11]. EMT is characterized by decreased expression of epithelial E-cadherin (encoded by the *CDH1* gene) and increased level of mesenchymal N-cadherin (encoded by the *CDH2* gene), which consequently leads to a reduced CDH1/CDH2 ratio. Several studies showed that EMT promotes metastasis in PCa [12]. In PCa, CC chemokine ligands (CCLs) are central regulators for inflammatory pathways [13,14]. The chemokine CCL2 was described to induce proliferation and migration of PCa cells [15]. Angiogenesis and invasion are two major carcinogenic processes, which are both controlled by matrix metalloproteinases (MMPs) [16,17]. Increased production of MMP7 and MMP9 was associated with malignant progression of PCa [18].

To study the effect of diabetes on the progression of PCa, we examined benign prostate (BEN) and PCa tissues of PCa patients with and without T2D. Furthermore, to replicate hyperglycemia in vitro, PC3 prostate adenocarcinoma cells were treated with increasing glucose concentrations. The expressions of candidate genes involved in EMT (*CDH1* and *CDH2*), inflammatory pathways (*CCL2* and *CCL5*) and invasion (*MMP7*, *MMP9* and *MMP14*), were measured using real time qPCR both in human prostate tissues and PC3 cells.

## 2. Experimental Section

### 2.1. Human Samples

**Figure 1:**
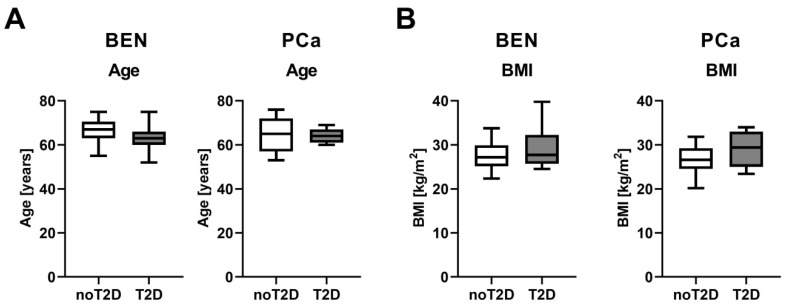
Benign prostate (BEN) (n = 17–17) and prostate cancer (PCa) (n = 11–11) tissues of PCa patients with (T2D) and patients without (noT2D) type 2 diabetes were studied. Age (**A**) and BMI (**B**) data are shown as Tukey box plots. Statistical significance was calculated using Mann–Whitney tests and considered as *p* < 0.05. No statistical significant differences were found.

Newly diagnosed PCa patients without prior anti-cancer therapy were recruited prior to radical prostatectomy. Tissue sampling was performed by an experienced uropathologist (M.S.) and all prostate cancers were classified as adenocarcinoma. PCa as well as benign tissues were immediately snap-frozen in liquid nitrogen and stored at −80 °C. For histological confirmation, hematoxylin and eosin staining was performed on paraffinized samples. Patient cohorts were age- and BMI-matched and n = 17–17 benign and n = 11–11 tumor tissue samples from patients with and without type 2 diabetes were included (Figure 1), as recently described [5]. Most of the patients with T2D received anti-diabetic medication (Table 1). To analyze PCa at similar tumor stages, all PCa samples had a Gleason score of 7a and 7b. Informed written consent was obtained from all participants, and the Ethics Committee of the University of Tübingen (575/2018BO2) approved the protocol in accordance with the Declaration of Helsinki. Total RNA was isolated using Allprep RNA/DNA/protein kit (Qiagen, Hilden, Germany) in accordance with the manufacturer’s description.

### 2.2. Cell Culture

The human prostate adenocarcinoma cell line PC3 was purchased from CLS-Cell line services GmbH (Eppelheim, Germany). Cells were maintained in low glucose (5.5 mM) DMEM (Gibco/Thermo Fisher Scientific, Karlsruhe, Germany) supplemented with 5% fetal bovine serum (FBS) (Bio&Sell, Feucht, Germany) [19]. One day before treatment, medium was changed to DMEM with 0.2% FBS. Following the addition of medium containing 5.5, 11.25 or 17.5 mM glucose, the cells were incubated for a further 72 h. There were five independent experiments performed. Following cell harvest, total RNA was isolated using Allprep DNA/RNA/miRNA kit (Qiagen) in accordance with the manufacturer’s instructions.

### 2.3. Quantitative Real-Time PCR

From total RNA, cDNA was synthesized using Transcriptor First Strand cDNA synthesis kit (Roche, Basel, Switzerland). Quantitative real-time PCRs were performed with LightCycler 480 Probes Master (Roche) and universal probe library probes using LightCycler 480 (Roche) as published previously [20]. Delta-delta crossing-point (Cp) values were calculated and the data were normalized to the housekeeping gene ubiquitin c (*UBC*) [21]. For real-time PCR analysis, the following primers and probes were applied: E-cadherin (*CDH1*) 5′-GCCGAGAGCTACACGTTCA and 3′-GACCGGTGCAATCTTCAAA (probe nr: 80), N-cadherin (*CDH2*) 5′-CTCCATGTGCCGGATAGC and 3′-CGATTTCACCAGAAGCCTCTAC (probe nr: 74), *MMP7* 5′-CGGATGGTAGCAGTCTAGGG and 3′-AGGTTGGATACATCACTGCATTAG (probe nr: 6), *MMP9* 5′-TCTTCCCTGGAGACCTGAGA and 3′-GAGTGTAACCATAGCGGTACAGG (probe nr: 27), *MMP14* 5′-CAGGAATGAGGATCTGAATGG and 3′-CCGAGGGGTCACTGGAAT (probe nr: 45), *CCL2* 5′-AGTCTCTGCCGCCCTTCT and 3′-GTGACTGGGGCATTGATTG (probe nr: 40), *CCL5* 5′-CCTCATTGCTACTGCCCTCT and 3′-GGTGTGGTGTCCGAGGAATA (probe nr: 16), *cJUN* 5′-AGGATAGTGCGATGTTTCAGG and 3′-GACTTCTCAGTGGGCTGTCC (probe nr: 65), *UBC* 5′-GGAAGGCATTCCTCCTGAT and 3′-CCCACCTCTGAGACGGAGTA (probe nr: 11), *PCNA* 5′-TGGAGAACTTGGAAATGGAAA and 3′-GAACTGGTTCATTCATCTCTATGG (probe nr: 69), *Vimentin* 5′-AGATGGCCCTTGACATTGAG and 3′-CAGGGAGGAAAAGTTTGGAA (probe nr: 11).

### 2.4. Statistical Analysis

For the gene expression analysis of PC3 data, paired Friedman test with Dunn’s correction for multiple comparisons were calculated using GraphPad Prism 8.4 (San Diego, CA, USA). For the gene expression analysis of human data, Mann–Whitney tests were applied using GraphPad Prism. Prior to regression analyses, data were logarithmic transformed to approximate normal distribution. Multiple linear regression models were built using JMP 14.2 (SAS, Cary, NC, USA). For human data, linear regression models were performed with adjustment for age and BMI. Statistical significance was considered as *p* < 0.05.

## 3. Results

To investigate the impact of diabetes on the progression of PCa, we analyzed benign prostate (BEN) and PCa tissues of age and BMI matched PCa patients with and without T2D (Figure 1). The following four groups were studied: BEN, noT2D (n = 17); BEN, T2D (n = 17); PCa, noT2D (n = 11) and PCa, T2D (n = 11).

**Figure 2:**
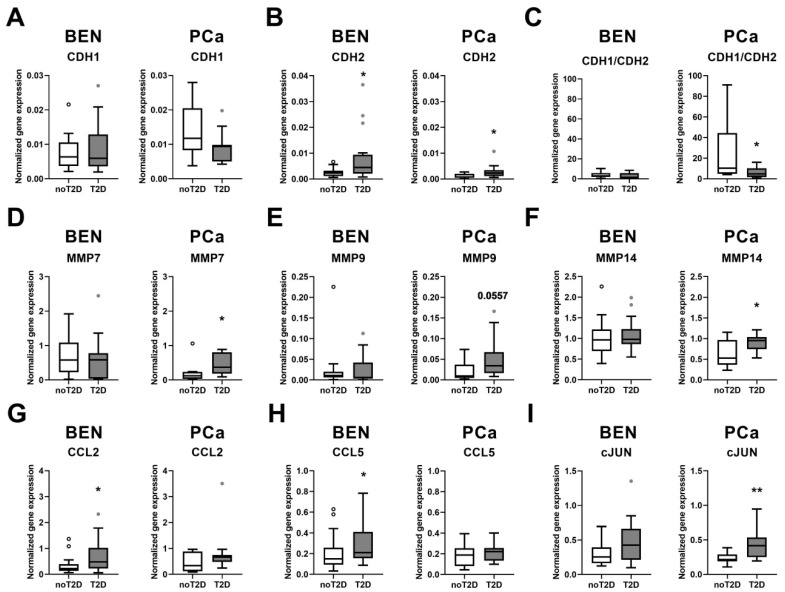
Gene expression levels of indicated genes were quantified in benign prostate (BEN) (n = 17–17) and prostate cancer (PCa) (n = 11–11) tissues of PCa patients with (T2D) and patients without (noT2D) type 2 diabetes. (**A**) CDH1, (**B**) CDH2, (**C**) CDH1/CDH2 ratio, (**D**) MMP7, (**E**) MMP9, (**F**) MMP14, (**G**) CCL2, (**H**) CCL5, (**I**) cJUN. Data are shown as Tukey box plots. White and grey dots denote individual values, which were higher than the sum of the 75th percentile plus 1.5-times inter-quartile range. Statistical significance was calculated using Mann–Whitney tests and considered as *p* < 0.05. * *p* < 0.05, ** *p* < 0.01. *p*-value for MMP9 PCa data is indicated.

Since epithelial-mesenchymal transition (EMT), invasion, and inflammation are major drivers of PCa carcinogenesis, we measured the transcript levels of the major factors regulating EMT (E-cadherin (*CDH1*) and N-cadherin (*CDH2*)), invasion (MMPs), and inflammation (CCLs). The gene expression level of *CDH1* was not significantly different among the analyzed groups (Figure 2A). Even though patients with T2D displayed higher *CDH2* mRNA levels in both BEN and PCa groups, the CDH1/CDH2 ratio was only decreased in PCa tissue of the T2D group when compared to patients without T2D (Figure 2B,C). An activated EMT is reflected by a reduced CDH1/CDH2 ratio [12]. EMT in PCa is additionally characterized by increased mesenchymal markers including vimentin [11]. By linear regression analyses we also found a significant negative association between the transcript level of vimentin and CDH1/CDH2 ratio in human prostate tissues (standardized beta = −0.4049; *p*-value = 0.0039), highlighting the switch in EMT in these samples. The transcript levels of *MMP7* and *MMP14* were significantly elevated in PCa tissue of patients with T2D, and in this group we also found a tendency for increased MMP9 levels (*p* = 0.0557) (Figure 2D–F). In BEN tissue of patients with T2D, we observed higher transcript levels of *CCL2* and *CCL5* (Figure 2G,H). The higher expression of *MMP* genes in PCa samples of patients with T2D and the increased *CCL* mRNA levels in BEN tissue of patients with T2D suggest that these genes could be induced by hyperglycemia. The mRNA level of *cJUN*, which is a key transcription factor driving carcinogenesis in PCa [22], was elevated in PCa samples of patients with T2D (Figure 2I). This result indicates that in PCa patients with T2D, the diabetes status could elevate the transcript of *cJUN*.

We recently observed an elevated mRNA level of the proliferation marker proliferating cell nuclear antigen (*PCNA*) [23] in PCa samples from patients with T2D [5]. To further evaluate the clinical relevance of the invasion and inflammatory markers analyzed in this study, the gene expression levels of these markers were correlated with the transcript levels of the *PCNA*. A linear regression model revealed a positive association between the transcript levels of *PCNA* and *MMP14* (Table 2) suggesting that invasive processes are linked to proliferative pathways in human prostate glands.

**Figure 3:**
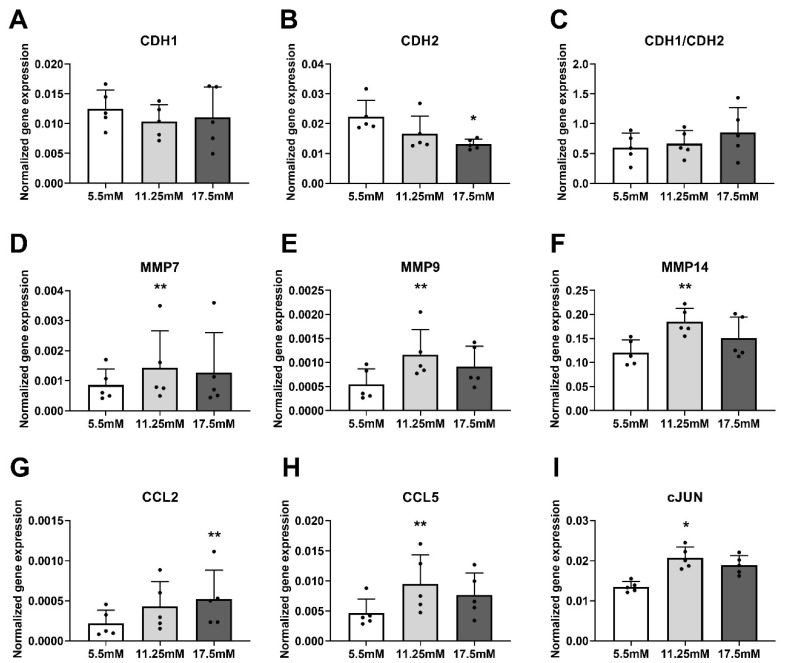
Gene expressions were quantified in 5.5, 11.25 and 17.5 mM glucose-treated PC3 cells (n = 5 independent experiments). (**A**) CDH1, (**B**) CDH2, (**C**) CDH1/CDH2 ratio, (**D**) MMP7, (**E**) MMP9, (**F**) MMP14, (**G**) CCL2, (**H**) CCL5, (**I**) cJUN. Statistical significance was compared to 5.5 mM control samples using paired Friedman test with Dunn’s correction for multiple comparisons. Dots denote individual experimental values. Statistical significance was considered as *p* < 0.05 and indicated as * *p* < 0.05; ** *p* < 0.01.

To assess whether hyperglycemia per se is able to modify the gene expression levels of EMT, invasion, and inflammatory markers, PC3 adenocarcinoma cells were treated with increasing glucose concentrations. In PC3 cells, hyperglycemic conditions did not alter the expression of *CDH1* and CDH1/CDH2 ratio, however *CDH2* showed lower expression level (Figure 3A–C). Nevertheless, increasing glucose concentrations induced the transcript levels of *MMP7, MMP9, MMP14, CCL2, CCL5* and *cJUN* in PC3 cells (Figure 3D–I). These data indicate that elevated blood glucose due to coexistent T2D may induce invasive and inflammatory markers in prostate cancer.

**Figure 4:**
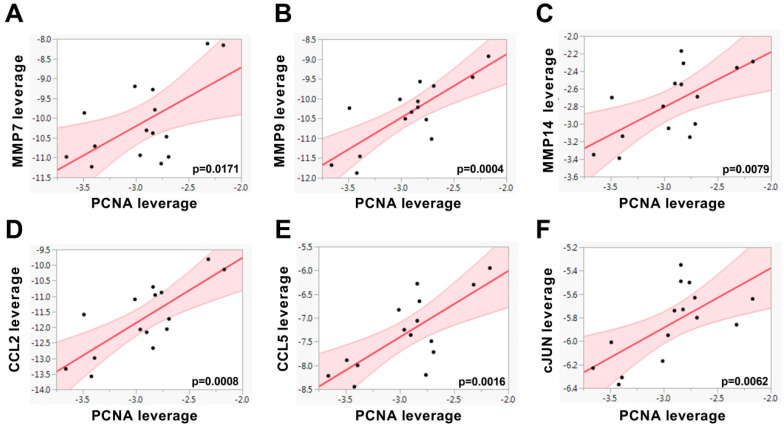
The gene expression level of *PCNA* was associated with the expression of (**A**) MMP7, (**B**) MMP9, (**C**) MMP14, (**D**) CCL2, (**E**) CCL5 and (**F**) cJUN using multiple linear regression models in 5.5, 11.25 and 17.5 mM glucose-treated PC3 cells (n = 15) and shown as leverage plots. Statistical significance was considered as *p* < 0.05. *p*-values are indicated in the figures.

Furthermore, in PC3 cells, linear regression analyses demonstrated that the gene expression level of the proliferation marker *PCNA* was positively associated with the mRNA levels of *MMP7, MMP9, MMP14, CCL2, CCL5* and *cJUN* (Figure 4). These results indicate that in PC3 cells, the invasive and inflammatory markers are linked to proliferative processes under hyperglycemic conditions.

## 4. Discussion

Accumulating data indicate that patients with T2D develop more aggressive prostate tumors than men without T2D [6,7,24]. Furthermore, among men with diabetes undergoing radical prostatectomy, an elevated HbA1c value was associated with a higher risk for metastatic PCa [25]. In line, PCa patients with concomitant T2D were also characterized by increased mortality [8,9,26,27,28]. Although the association between advanced PCa and T2D is already reported, the underlying molecular mechanisms are poorly understood. To assess the impact of diabetes on the development and progression of PCa, we examined human benign prostate (BEN) and PCa samples of PCa patients with and without T2D. Furthermore, to replicate the effect of hyperglycemia in vitro, PC3 prostate adenocarcinoma cells were treated with increasing glucose concentrations.

In this study, we focused on key regulators, which are involved in the development of diabetes and diabetic complications. Inflammatory processes are intimately interconnected with the development of insulin resistance as well as diabetes and several chemokines are major regulators of inflammation [29]. The chemokines CCL2 (also known as MCP-1) and CCL5 (also known as RANTES) are produced by immune cells, endothelial cells, pancreatic islets and adipocytes and they coordinate inflammatory cell infiltration including macrophages, dendritic cells and T cells [29,30]. The circulating level of CCL2 is correlated with insulin resistance and T2D [31]. A recent study investigated potential serum biomarkers for diabetes and observed CCL5 among the nine identified serum proteins [32]. Matrix metalloproteinases (MMPs) are implicated in various diabetic complications including retinopathy, vasculopathy, and diabetic foot [33,34]. Small molecules, which inhibit MMPs, show promising results leading to accelerated wound healing in diabetic rodent models [35].

We detected higher expression of the investigated *MMPs* and *CCLs* in human prostate samples from patients with T2D as well as in PC3 cells exposed to high glucose. The detailed mechanism for the upregulation of these genes is still not completely understood. Recent data suggest that hyperglycemia could be involved in the induction of *MMP* and *CCL* genes. In streptozotocin-treated mice, which is a rodent model for insulin deficient type 1 diabetes [36], high glucose increased the expression of renal *CCL2* and *CCL5*, which was associated with the induction of diabetic nephropathy [37]. In diabetic NOD (nonobese diabetes) mice, another type 1 diabetes rodent model, Aaron-Brooks and colleagues reported elevated *MMP* and *CCL* expressions in the prostate compared with non-diabetic animals [38]. These results indicate that the hyperglycemic condition could be the missing underlying mechanism for the increased expression of *MMP* and *CCL* genes. In line, poorer prognosis was reported in men with PCa and elevated blood glucose [39,40]. The analyzed patients with T2D received various anti-diabetes drugs before prostatectomy (Table 1). Therefore, the observed prostatic gene expression pattern in T2D patients could eventually be influenced by anti-diabetic therapy, which we could not specifically test in our study.

**Figure 5:**
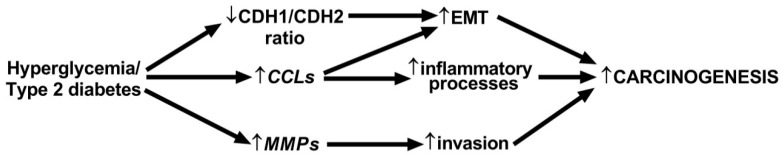
Proposed carcinogenic mechanism of the observed changes at transcript levels in prostate tissue of patients with type 2 diabetes. CDH1: E-cadherin; CDH2: N-cadherin; EMT: epithelial-mesenchymal transition; CCLs: CCL chemokine ligands; MMPs: matrix metalloproteinases.

In addition to their role in diabetes, MMPs and CCLs are known to drive carcinogenesis. Although some authors observed that specific MMPs may have protective roles in cancer progression, many reports described the importance of MMP activation in carcinogenesis [41]. For PCa, several studies demonstrated an association of increased production of MMPs including MMP7, MMP9 and MMP14 with malignant progression of PCa [18]. MMPs are involved in various carcinogenic processes including angiogenesis, differentiation, proliferation and apoptosis [16,42]. The most prominent carcinogenic effect of MMPs is the degradation of the extracellular matrix components of the basement membrane, which is crucial for invasion and a consecutive metastatic process [18,42]. Therefore, it is conceivable that hyperglycemia accelerates carcinogenesis and worsens prognosis of PCa patients with T2D. The higher expression of *MMP* transcripts in PCa samples of patients with T2D indicates that MMPs could induce the invasive capacity of PCa and in turn may drive metastasis in patients with diabetes (Figure 5).

In addition to the role of MMPs in ECM remodeling, MMPs were shown to modify non-matrix substrates such as inflammatory cytokines and chemokines [43,44]. Furthermore, MMP7 and MMP9 could potentially cleave CCL2 and CCL5. However, the biological consequences of these cleavages are yet to be determined [45,46]. Nevertheless, inflammation is a crucial step in cancer development and recruitment of immune cells to the primary cancer site is a hallmark of PCa [47]. Chemokines including CCL2 and CCL5 are described to attract tumor-associated macrophages and monocytes during this infiltration process [47]. These chemokines interact with the androgen receptor (AR) signaling and current data suggest that CCL5 is an upstream regulator of AR, however CCL2 is reported to be downstream of AR [48]. In this study, benign prostate tissues of patients with T2D showed elevated expressions of *CCL2* and *CCL5* than samples of patients without diabetes. Given the role of CCLs in the inflammation of PCa, these data suggest that the higher transcript levels of *CCLs* contribute to the inflammation of the prostate in patients with T2D (Figure 5). Since in PCa samples of patients with T2D we did not observe increased *CCL* expressions, our data point to a potentially relevant impact of inflammatory processes in the early phase of carcinogenic alterations in prostate tissue.

In the progression of PCa, epithelial-mesenchymal transition (EMT) has a crucial role [49]. EMT transition is characterized by a decrease in E-cadherin (*CDH1*) and an increase in N-cadherin (*CDH2*) levels demonstrated by a reduced CDH1/CDH2 ratio [12]. In PCa samples of patients with T2D, we found a lower CDH1/CDH2 ratio suggesting an activated EMT. The transcription factor cJUN has been shown to initiate EMT migration and invasion of PCa cells [12]. The elevated mRNA level of *cJUN* in PCa tissues of patients with T2D suggests that this transcription factor may activate EMT in these samples. In addition, in PC3 cells, high glucose treatment enhanced insulin-driven EMT [50], suggesting an additional role of hyperglycemia in the activation of EMT in patients with T2D. Furthermore, the secretion of chemokines and EMT are interconnected, since the activation of CCL2 was reported to induce EMT [47]. Here we observed a decreased CDH1/CDH2 ratio in PCa samples of patients with T2D suggesting that EMT is induced in these samples, which may contribute to the more aggressive PCa phenotype of patients with diabetes (Figure 5).

Recent studies indicate that at least a part of the used pharmacotherapy for diabetes treatment may have unrecognized potential also for the treatment of cancer [51]. The most commonly prescribed anti-diabetic drug for type 2 diabetes, metformin, is believed to have anti-cancer effects in several cancer types including PCa [51]. Metformin use was also associated with improved survival for patients with advanced PCa on androgen deprivation therapy [52]. Furthermore, Taussky and colleagues analyzed 48 months survival in patients with or without diabetes and found that metformin users had a better survival ratio than patients with diabetes not taking metformin [53]. The beneficial anti-cancer effects of metformin could be at least partly attributed to the fact that metformin inhibits EMT of PCa in preclinical studies [49]. These results underline that patients who suffer from both T2D and PCa may benefit from metformin treatment. Although most reports observed anti-cancer potential for metformin, several randomized trials did not find beneficial effects of metformin for important clinical parameters of cancer, including progression-free survival and overall survival [54,55,56]. Some studies even reported pro-tumorigenic activity for metformin and suggested that metformin might induce pro-tumorigenic macrophage phenotype, and increase angiogenesis and tumor growth acceleration [57,58,59]. In July 2020, the potential carcinogenic N-nitrosodimethyl amine, was measured at higher than allowed concentrations in some commercial metformin preparations [60]. Therefore, several pharmaceutical companies voluntary recalled metformin products (https://www.fda.gov/drugs/drug-safety-and-availability/fda-updates-and-press-announcements-ndma-metformin, accessed on 6 November 2020). Hence, the impact of metformin on PCa needs to be clarified in future studies. They should address the entirety of the effects of metformin on cancer progression and test the clinical benefit of metformin in the appropriate randomized trials.

## 5. Conclusions

In prostate tissue of patients with T2D, we observed a decreased CDH1/CDH2 ratio, and higher *CCL* and *MMP* transcripts. Furthermore, high glucose treatment of PC3 cells led to increased expression of *CCL* and *MMP* genes. These results therefore indicate (Figure 5) that hyperglycemia may induce EMT, inflammatory processes, and enhance invasive capacity leading to accelerated PCa carcinogenesis in patients with T2D.

## Figures and Tables

**Table 1:** Most patients with T2D were treated with antidiabetic medications. Numbers (nr) denote the number of patients, who received the respective treatment.

T2D Therapy	T2D Patients with BEN Samples	T2D Patients with PCa Samples
Insulin	2	1
Metformin	10	6
Repaglinid	2	1
Glimepiride	0	1
Acerbose	1	0
Sitagliptin	0	1
Diet modification only	6	4
Total nr	17	11

**Table 2:** The gene expression level of proliferating cell nuclear antigen (*PCNA*) was associated with the indicated genes using linear regression models adjusted for age and BMI in human prostate tissues (n = 56). Standard beta values represent standardized regression coefficients. Statistical significance was considered as *p* < 0.05.

	*PCNA*	
Gene	Standard Beta	*p*-Value
*MMP7*	0.1052	0.4695
*MMP9*	0.2007	0.1692
*MMP14*	0.5093	<0.0001
*CCL2*	0.0869	0.5552
*CCL5*	0.1882	0.1862
*cJUN*	0.1542	0.2453
